# Measuring Nasal Airway Resistance to Personalize Surgery for Nasal Obstruction in OSA Patients

**DOI:** 10.3390/jpm15120608

**Published:** 2025-12-08

**Authors:** Giuseppe Lunardi, Francesco Giombi, Gian Marco Pace, Michele Cerasuolo, Giuseppe Spriano, Luca Malvezzi

**Affiliations:** 1 Otorhinolaryngology Head & Neck Surgery Unit, Casa di Cura Humanitas San Pio X, Via Francesco Nava 31, 20159 Milan, Italy; giuseppe.lunardi@sanpiox.humanitas.it (G.L.); michele.cerasuolo@sanpiox.humanitas.it (M.C.); 2 Otorhinolaryngology Unit, IRCCS Humanitas Research Hospital, Via Manzoni 56, 20089 Rozzano, Italy; gianmarco.pace@humanitas.it (G.M.P.); giuseppe.spriano@hunimed.eu (G.S.); luca.malvezzi@humanitas.it (L.M.); 3 Department of Biomedical Sciences, Humanitas University, Via Rita Levi Montalcini 4, 20090 Pieve Emanuele, Italy

**Keywords:** septoplasty, obstructive sleep apnea, rhinomanometry, nasal obstruction, sleep endoscopy

## Abstract

**Objective:** This study aimed to measure nasal airway resistance (NAR) in obstructive sleep apnea (OSA) patients with nasal obstruction using active anterior rhinomanometry (AAR) and to evaluate whether NAR can predict the indication to include septoplasty as an additional procedure alongside drug-induced sleep endoscopy (DISE) and inferior turbinoplasty. **Methods**: We performed a retrospective observational study in OSA patients with nasal obstruction. According to nasal endoscopy and CT findings, patients were planned for either DISE with inferior turbinoplasty alone or DISE with inferior turbinoplasty and septoplasty. All patients underwent preoperative AAR, carried out under baseline and post-decongestion conditions. To test the ability of NAR to predict septoplasty indication, receiver operating characteristic (ROC) curves were generated for baseline and post-decongestion values. Logistic regression combined inspiratory/expiratory and unilateral/total NAR. The Area Under the Curve (AUC) was used to evaluate diagnostic accuracy, and optimal cut-offs were identified using Youden’s index (J). **Results**: Forty-eight patients were included. Baseline NAR showed low accuracy (median AUC: 0.540 unilateral, 0.562 total) and no valid cut-offs were identified (median J: 0.213 unilateral, 0.233 total). Post-decongestion NAR performed better (median AUC: 0.649 unilateral, 0.738 total). Inspiratory and expiratory unilateral values merged with binary regression improved prediction (AUC 0.677 and 0.709). The highest accuracy was achieved when all rhinomanometric parameters were integrated into one logistic model (AUC = 0.947). **Conclusions**: Preoperative AAR may help refine nasal surgical planning during DISE in OSAS patients, supporting a personalized approach and potentially reducing the need for staged nasal procedures.

## 1. Introduction

Nasal obstruction is a common condition characterized by increased resistance to nasal airflow, inspiratory or expiratory. It may result from anatomical defects (e.g., septal deviation, nasal valve collapse, neoplasms) or reversible functional disorders (e.g., hypertrophic rhinitis, rhinosinusitis) [1]. Regardless of cause, it can significantly affect quality of life and burden healthcare systems [2,3]. Although upper airway (UA) collapse in obstructive sleep apnea syndrome (OSAS) typically occurs at the level of the soft palate and the tongue base [4], growing evidence indicates that the sinonasal compartment also plays a relevant role in the pathophysiology of sleep-disordered breathing [5]. Increased nasal resistance can promote a shift from nasal to oral breathing during sleep, which alters upper airway aerodynamics, reduces pharyngeal stability, and can favor collapse. Mouth breathing is associated with reduced negative pressure reflex activation and a posterior displacement of the tongue, both of which may contribute to airway obstruction [6]. In this context, improving nasal patency—whether through medical therapy or surgical correction—has been shown to decrease inspiratory resistance, improve subjective sleep quality, and lead to modest reductions in OSAS severity, even if it rarely eliminates the disorder entirely [7,8]. With the growing awareness of the prevalence and clinical impact of OSAS, an increasing number of patients are now referred for structured diagnostic workup and treatment of sleep-disordered breathing [9]. Because of the inherently multidisciplinary nature of this condition, otolaryngologists, alongside other specialists, play a key role in both diagnostic evaluation and therapeutic planning.

Within this context, drug-induced sleep endoscopy (DISE) has become a critical step in the evaluation of the obstructive upper airway [10]. Although primarily a diagnostic procedure, DISE requires sedation, which provides an opportunity to address nasal obstruction surgically during the same session in selected patients. Performing nasal surgery concomitantly with DISE can reduce the need for staged interventions; however, a personalized approach is essential to maximize efficacy and avoid standardized, potentially suboptimal strategies. For instance, some centers routinely combine DISE with inferior turbinoplasty (IT) regardless of the underlying anatomical or functional pattern of obstruction [11]. This “one-size-fits-all” approach may be insufficient in patients with complex nasal airflow limitation, such as significant septal deviation. Given the well-documented impact of nasal obstruction on quality of life, which further compounds the burden of OSAS, nasal surgery should be precisely tailored rather than generic. Beyond clinical examination, nasal endoscopy, and imaging, functional assessment tools can help refine surgical planning. Among these, anterior active rhinomanometry (AAR), performed both under baseline conditions and after topical decongestion, may help differentiate predominantly mucosal versus structural obstruction [12]. Such information could guide the intraoperative decision between isolated IT and combined septoplasty with IT during DISE, potentially improving surgical accuracy and reducing the need for subsequent staged nasal procedures. The present study aimed to measure nasal airflow resistance (NAR) values in obstructive sleep apnea (OSA) patients with nasal obstruction using active anterior rhinomanometry (AAR), and, through a retrospective analysis of our cohort, to assess whether preoperative rhinomanometric parameters could have provided useful guidance in selecting the most appropriate nasal surgical strategy at the time of DISE.

## 2. Materials and Methods

This single-center retrospective observational study was conducted at Casa di Cura Humanitas San Pio X (Milan, Italy) between January 2023 and January 2024. Eligible participants were adults (≥18 years) with a diagnosis of obstructive sleep apnea (OSA) confirmed by HSAT, and nasal obstruction. All patients were referred for DISE and underwent concomitant nasal surgery for respiratory obstruction (either inferior turbinoplasty or septoplasty with inferior turbinoplasty). Each patient completed a preoperative workup including ENT examination with nasopharyngoscopy, and a head computed tomography (CT) scan. According to endoscopic and radiological findings, patients were assigned either to septoplasty plus inferior turbinoplasty performed in conjunction with DISE or to DISE with inferior turbinoplasty alone.

As a part of the preoperative assessment, all patients were required to undergo AAR. The decision regarding the type of surgery was performed independently of the rhinomanometric results.

All surgical procedures were carried out under a day-hospital regimen. Only Caucasian patients were included. We defined this exclusion criterion to limit selection bias related to anatomical variability. Because the vast majority of patients evaluated in our rhinomanometry service were Caucasian, stratification by ethnicity would have resulted in insufficient statistical power. Therefore, we excluded non-Caucasian patients to control for known ethnic differences in nasal physiology, including the reference intranasal pressure of 150 Pa (which is less consistently achieved in Asian patients) and small but documented variations in mean nasal resistance across populations. These variations are likely related to differences in the minimal cross-sectional area and in nasal anatomy (e.g., the nostrils, the shape, or the piriform aperture) [13,14,15,16,17]. This approach ensured a more homogeneous cohort and strengthened the generalizability of our findings within the Caucasian population.

Patients with dynamic alar cartilage collapse during inspiration (i.e., nasal valve collapse) or with extensively pneumatized concha bullosa (Bolger classification) [18] were excluded. Home Sleep Apnea Testing was performed using an Embletta^®^ Multi Parameter Recorder-Polygraph (RemLogicE^®^ 3.4.1, Embla Systems, Kanata, ON, Canada). The recorded parameters were as follows: the Apnea–Hypopnea Index (AHI), the Oxygen-Desaturation Index (ODI), the mean oxygen saturation (Mean SpO_2_), the lower oxygen saturation level (Min SpO_2_), and the relative time of sleep spent with saturation levels < 90% (CT90). Consistent with the American Academy of Sleep Medicine (AASM) guidelines [19], apnea was defined as a cessation of airflow lasting at least 10 s, while hypopnea was defined as a reduction in airflow amplitude of at least 80% for a duration of at least 10 s. Thresholds for OSA severity were defined as follows: 5–14.9 events/hour, mild OSA; 15–29.9 events/hour, moderate OSA; and ≥30 events/hour, severe OSA. Oxygen saturation was monitored using a pulse oximeter.

Active anterior rhinomanometry was performed using the Rinopocket ED200 system™ (Medicare Solutions, Bologna, Italia). To ensure reproducibility, all measurements were conducted under stable environmental conditions, with room temperature maintained between 22 °C and 23 °C and constant humidity levels [20]. Prior to examination, patients were instructed to remain seated for at least 15 min. The procedure was then performed in a semi-recumbent position with an inclination of 15–30° (low Fowler position). In accordance with standard clinical practice, nasal airflow resistance was assessed separately for each nostril during both inspiratory and expiratory phases, both at baseline and after administration of a topical nasal decongestant (0.1% xylometazoline hydrochloride). Paradoxical responses were defined by increased nasal airway resistance > 20% after administration of a topical decongestant [21].

The nasal airway resistance values were determined for each nostril based on the slope of the pressure–volume curves analyzed at 150 Pa and expressed in Pa/cm^3^/s, following current guidelines [14]. Total inspiratory and expiratory NAR were ultimately recorded. Cut-off values were defined based on current guidelines: 0.500 Pa/cm^3^/s for unilateral and 0.250 Pa/cm^3^/s for total nasal resistances, respectively [13,22].

Statistical analysis was performed using IBM^®^ SPSS Software for Macintosh, Version 26.0 (IBM Corp., Armonk, NY, USA). Dichotomous variables were expressed by relative number and percentage. Continuous parametric variables were expressed through mean ± standard deviation (sd) and range. A power analysis was conducted assuming a 95% confidence level and an expected proportion of OSA patients with pathological nasal airflow resistances of 7%, based on previously reported prevalence data on existing literature [23]. The margin of error, which is an estimate of random sampling error, was assessed as a measure of the adequacy of the sample size. Receiver operating characteristic (ROC) curves were calculated to assess the accuracy of rhinomanometry in predicting the indication to septoplasty in the course of DISE. The interpretation of the Area Under the Curve (AUC) was adherent to the classification proposed by Swets [24], which defines the diagnostic accuracy of a test as follows:

1. AUC = 0.5: The test provides no diagnostic information;
2. 0.5 < AUC ≤ 0.7: The test has low accuracy;
3. 0.7 < AUC ≤ 0.9: The test has moderate accuracy;
4. 0.9 < AUC < 1.0: The test has high accuracy;
5. AUC = 1.0: The test is considered perfect.

Receiver operating characteristic curves were first calculated considering unilateral and total NAR separately, both at baseline and after nasal decongestion; further curves were then built merging NAR values through binary regression.

The Youden’s index (*J*) was calculated for each ROC curve, based on the following formula:

J=sensitivity+specificity−1

The point with the highest *J* value (range: 0–1) represented the best balance between sensitivity and specificity and was therefore considered the optimal cut-off.

A univariate binary logistic regression model was built to assess the correlation between clinically relevant septal deviation (indication for septoplasty) and NAR values.

## 3. Results

### 3.1. General Characteristics

A total of 48 mild- to severe-OSA patients were enrolled (males: 35/48, 72.9%; mean age: 53 ± 11 years, range 31–76). The mean AHI and ODI were 21.7 ± 15.0/h (range: 59.9/h–5.0/h) and 21.8/h ± 15 (range: 59.7/h–5.0/h). The mean and minimum SpO_2_ were 93.5 ± 2.0% (range: 90.0–96.9%) and 81.0 ± 7.0% (range: 68.0–93.9%), respectively. The mean CT90 was 8.9 ± 11.0% (range: 0.9–47.4%). All patients underwent DISE with concomitant nasal surgery (DISE with turbinoplasty = *n* = 27/48, 56.3%; DISE + septoturbinoplasty *n* = 21/48, 43.7%). The demographic characteristics and HSAT parameters are presented in Table 1.

In the power analysis, for an overall population of 48 patients, we observed a margin of error of 7.22%, assuming a 95% confidence level.

### 3.2. Nasal Airflow Resistances

Under basal conditions, the mean NAR was 0.401 ± 0.537 Pa/cm^3^/s and 0.364 ± 1.075 Pa/cm^3^/s for unilateral inspiratory and expiratory values, respectively. The mean total inspiratory and expiratory NAR at baseline were 0.183 ± 0.154 Pa/cm^3^/s and 0.178 ± 0.130 Pa/cm^3^/s.

After nasal mucosa decongestion, the mean inspiratory and expiratory NAR were reduced to 0.228 ± 0.330 Pa/cm^3^/s and 0.249 ± 0.734 Pa/cm^3^/s, respectively. The decongested mean total inspiratory and expiratory NAR were 0.103 ± 0.128 and 0.134 ± 0.108. Rhinomanometric parameters are presented in Table 2 for ease of reference.

We observed two (*n* = 2/48, 3.9%), paradoxical responses to nasal decongestant, coherently with the main reference studies [25].

### 3.3. ROC Analysis

**Figure 1:**
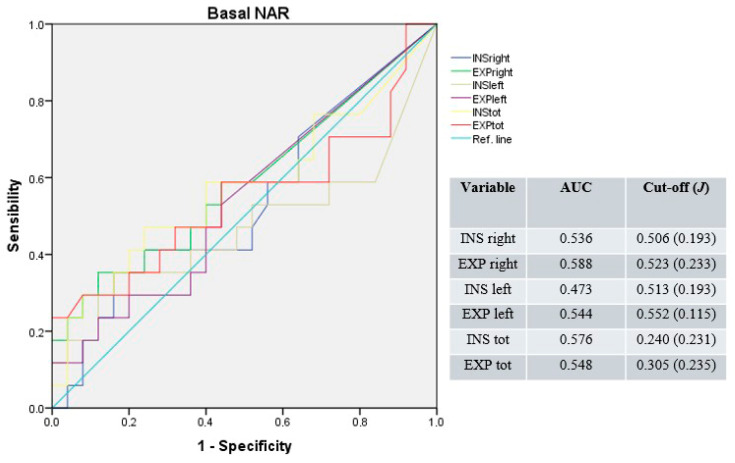
Receiver operating characteristic curves for unilateral and total NAR under basal conditions. Blue line: right inspiratory NAR; green line: right expiratory NAR; brown line: left inspiratory NAR; purple line: left expiratory NAR; yellow line: total inspiratory NAR; red line: total expiratory NAR; light-blue line: reference.

Under basal conditions, the median AUC was 0.540 (range: 0.473–0.588) for unilateral and 0.562 (range: 0.548–0.576) for total NAR values. The median cut-off was 0.518 Pa/cm^3^/s (range: 0.506–0.552; median J = 0.213) for unilateral and 0.273 Pa/cm^3^/s (range: 0.240–0.305; median *J* = 0.233) for total NAR values (Figure 1).

**Figure 2:**
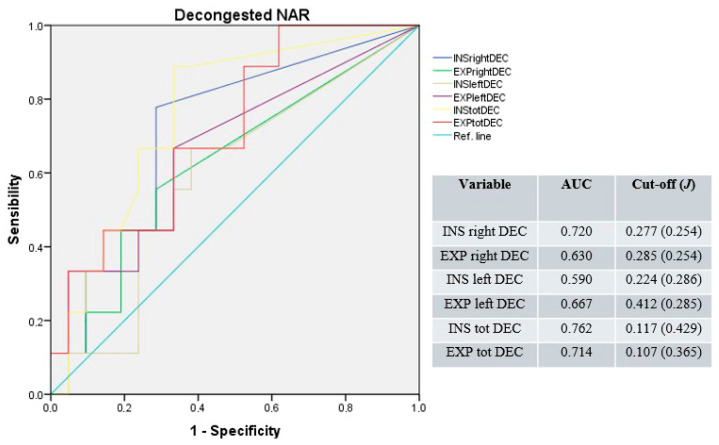
Receiver operating characteristic curves for unilateral and total NAR after nasal mucosa decongestion. Blue line: right inspiratory NAR; green line: right expiratory NAR; light-brown line: left inspiratory NAR; purple line: left expiratory NAR; yellow line: total inspiratory NAR; red line: total expiratory NAR; light-blue line: reference.

After nasal mucosa decongestion, the median AUC was 0.649 (range: 0.590–0.720) for unilateral and 0.738 (range: 0.762–0.714) for total NAR values. The median cut-off was 0.281 Pa/cm^3^/s (range: 0.224–0.412; median *J* = 0.254); for unilateral and 0.112 Pa/cm^3^/s (range: 0.107–0.117; median *J* = 0.397) for total resistances (Figure 2).

With univariate binary logistic regression, no statistically significant correlation was observed between the indication to septoplasty and single rhinomanometric values (unilateral and total inspiratory and expiratory NAR, both at baseline and after nasal mucosa decongestion; *p* > 0.05).

**Figure 3:**
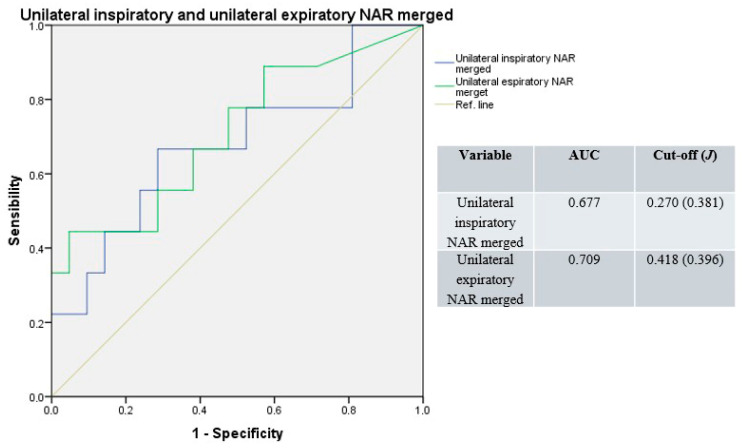
Receiver operating characteristic curves for unilateral resistances; basal and decongested NAR values merged. Blue line: unilateral inspiratory resistance; green line: unilateral expiratory resistance; light-brown line: reference.

Merging basal and decongested unilateral NAR values, the median AUC of the resulting ROC curve was 0.693 (range: 0.677–0.709), with a median cut-off of 0.344 Pa/cm^3^/s (range: 0.270–0.418; median *J* = 0.389; Figure 3).

**Figure 4:**
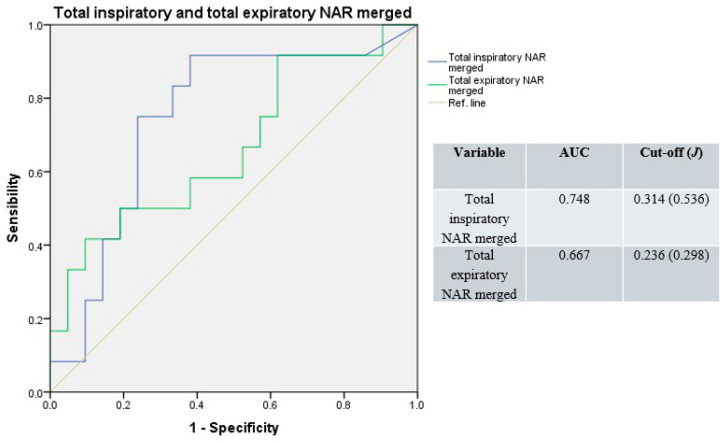
Receiver operating characteristic curves for total resistance; basal and decongested NAR values merged. Blue line: total inspiratory resistance; green line: total expiratory resistance; light-brown line: reference.

Similarly, merging basal and decongested total NAR values, the median AUC result was 0.708 (range: 0.667–0.748), with a median cut-off of 0.275 Pa/cm^3^/s (range: 0.236–0.314; median *J* = 0.417; Figure 4).

**Figure 5:**
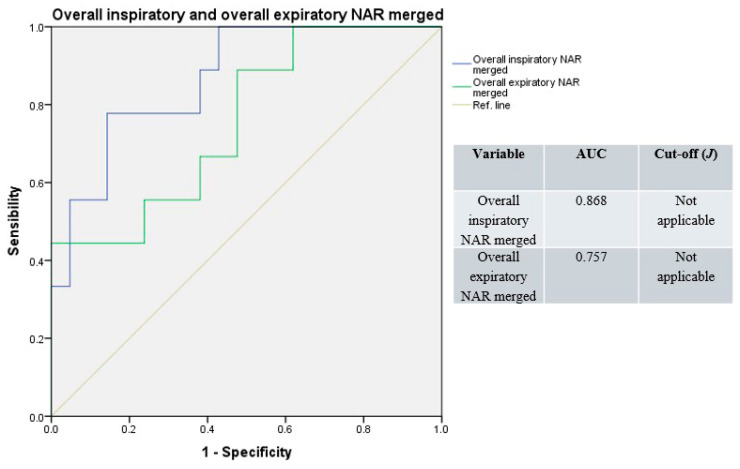
Receiver operating characteristic curves, overall (e.g., basal and decongested), unilateral, and total NAR values merged. Blue line: overall inspiratory resistances; green line: overall expiratory resistances; light-brown line: reference.

Receiver operating characteristic curves resulting from merged overall unilateral and total NAR values had good accuracy (median AUC = 0.813, range: 0.757–0.868; Figure 5), with a non-applicable Youden’s index (due to the merging of variables with different cut-offs).

**Figure 6:**
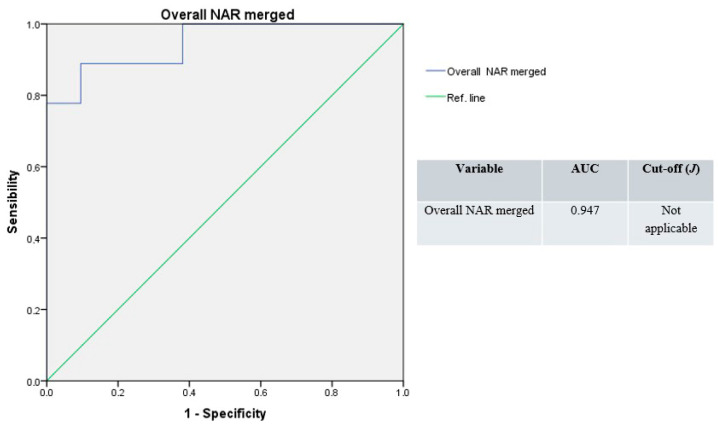
Receiver operating characteristic curves, overall NAR merged (blue line); green line: reference.

The ROC curve resulting from the whole NAR values merged produced the highest accuracy (AUC = 0.947), with a non-applicable Youden’s index (Figure 6).

## 4. Discussion

Nasal obstruction is a highly prevalent condition with a well-documented negative impact on patients’ quality of life, affecting sleep, daytime functioning, and overall well-being. Symptoms such as chronic congestion, impaired nasal breathing, and nocturnal discomfort can lead to fatigue, reduced cognitive performance, and decreased productivity, while also imposing a considerable burden on healthcare systems [26]. Previous studies have shown that nasal obstruction contributes to snoring and apneic episodes by favoring nocturnal mouth breathing, which facilitates posterior tongue retraction, reduces the retroglossal diameter, narrows the pharyngeal lumen, and ultimately promotes upper airway collapse [27,28]. The nose accounts for up to 50% of total airway resistance, and according to the Starling resistor model, upstream nasal obstruction generates downstream negative intraluminal pressure, increasing the risk of oropharyngeal collapse in predisposed individuals [29]. This effect is further accentuated in the supine position, where reflex-mediated increases in nasal resistance and passive venous congestion worsen airflow limitation. In addition, nitric oxide (NO), largely produced in the nasal and paranasal mucosa, has been implicated in maintaining upper airway patency by acting as an aerotransmitter between the nasal passages, pharyngeal musculature, and lungs. Reduced nasal airflow may therefore decrease NO delivery to the lower airways, increasing upper airway collapsibility and contributing to ventilation–perfusion mismatch [30].

Epidemiological studies have reported that septal deviation is up to four times more prevalent in patients with OSAS compared with the general population, further supporting the hypothesis that nasal structural abnormalities may predispose to or aggravate obstructive events [31]. Moreover, surgical correction of nasal obstruction, such as septoplasty or turbinate reduction, has been associated with improvements in polygraphic indices and subjective sleep quality, although these interventions rarely resolve OSAS on their own. Similarly to Fiorita et al. [11], Bican et al. observed a reduction in apneic episodes following nasal valve surgery [32]. A recent meta-analysis by Wu et al. found improved AHI and sleep-related symptoms after isolated nasal surgery [33]. Nasal obstruction is also clinically relevant because it impacts the efficacy of and adherence to CPAP therapy, which remains the gold standard for OSAS management. Impaired nasal airflow hinders the effective transmission of positive pressure to the collapsible pharyngeal airway, reduces patient comfort, and contributes to CPAP intolerance or discontinuation. Optimizing nasal patency can therefore enhance CPAP effectiveness and improve long-term adherence [34]. Collectively, these findings underscore the importance of a thorough assessment of nasal function during the preoperative evaluation of OSA patients. Nevertheless, to our knowledge, this is the first study that demonstrates the feasibility of a personalized approach to the preoperative workup.

Treating nasal obstruction is not only important for symptom relief and quality-of-life improvement but also acquires additional value in the comprehensive management of OSA patients. In the setting of DISE, where the upper airway is already being functionally assessed under sedation, combining tailored nasal surgery with diagnostic evaluation may streamline care and reduce the need for staged interventions. However, to maximize effectiveness, nasal surgery should be individualized rather than performed according to standardized algorithms. To date, the evaluation of nasal obstruction in OSA remains largely clinical. Nasal endoscopy and high-resolution CT have improved anatomical assessment, but validated methods for functional airflow evaluation are not yet routinely applied in practice. Objective functional assessment tools such as AAR, especially when performed both before and after decongestion, may provide useful data to distinguish structural from mucosal obstruction and guide the surgical strategy during DISE [25].

**Figure 7:**
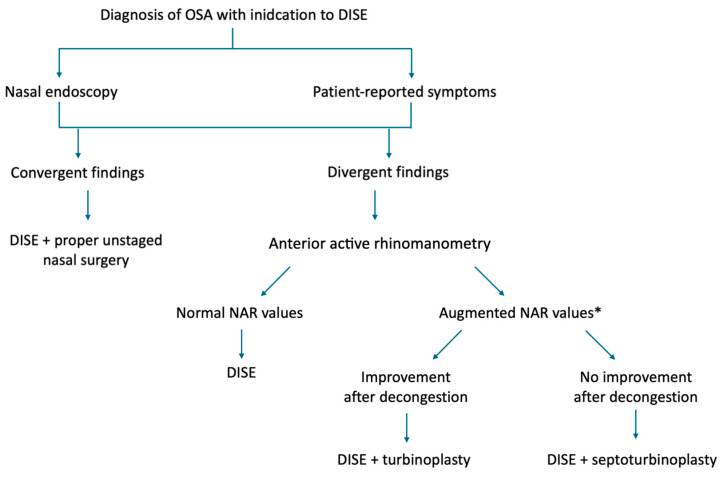
Flowchart describing the real-life surgical decision-making process. * suggested cut-offs: unilateral basal NAR = 0.518 Pa/cm^3^/s; total basal NAR = 0.273 Pa/cm^3^/s; after decongestion unilateral NAR = 0.281 Pa/cm^3^/s; after decongestion total NAR = 0.112 Pa/cm^3^/s.

In this retrospective study, we evaluated NAR derived from anterior active rhinomanometry AAR in a cohort of OSA patients with symptomatic nasal obstruction undergoing DISE with concomitant nasal surgery (either inferior turbinoplasty alone or combined septoplasty and inferior turbinoplasty). Our findings suggest that AAR can reliably support the selection of a tailored and potentially more effective surgical approach. Importantly, its diagnostic performance improved when multiple rhinomanometric parameters were analyzed together, while relying solely on baseline measurements was less informative. This is likely due to the confounding effect of mucosal hypertrophy, which was present across patients regardless of septoplasty indication. After topical nasal decongestion, the residual resistance values more accurately reflected the structural component of septal deviation, thereby enhancing the predictive value of AAR and increasing the reliability of surgical decision-making (Figure 7).

Notably, no significant correlation was found between the indication for septoplasty and either baseline or decongested NAR values in the binary logistic regression analysis. Although this finding may appear contradictory, it can be explained by the study design, as patients were assigned to either septoplasty or turbinoplasty alone based solely on clinical evaluation. In our opinion, this further supports the complementary role of AAR in the preoperative workup. Nevertheless, considering that in this specific limited population the underlying inflammatory processes varied across patients due to different upper airway comorbidities (Table 1), we believe that these results should be interpreted with caution, as preliminary exploratory data. Larger cohorts are required to minimize potential selection bias.

The utility of AAR in enhancing the management of nasal dysfunction has been previously reported by Holstrom et al., who demonstrated that incorporating rhinomanometric assessment into patient selection increased surgical success rates from 69% to 85% in those who had pathological NAR [35]. Moreover, the applicability of AAR has been recently demonstrated in cohorts of patients affected by chronic sinonasal conditions beyond septal deviation, including severe recalcitrant nasal polyposis requiring treatment with monoclonal antibodies [36]. Hereby, our results further support the role of AAR as a supportive tool to refine preoperative planning in patients with obstructive sleep apnea and clinically relevant nasal obstruction scheduled for DISE with concomitant nasal surgery. In particular, interpreting the overall patterns of NAR and adding post-decongestion measurements can help the surgeon weigh mucosal versus structural contributors to obstruction and select a more individualized nasal procedure. This approach has the potential to improve the precision of the nasal intervention and to reduce the likelihood of secondary staged procedures, without a change in the overall management strategy of OSA. A strength of this work is the use of an objective functional test within a real-world cohort, which enhances external relevance and suggests immediate clinical applicability where AAR is already available. It is also important to clarify that the indication for combined septoplasty and inferior turbinoplasty in our practice derived from endoscopic findings and cross-sectional imaging, while AAR formed part of the diagnostic pathway and was used to document function and, when available, to monitor postoperative evolution. This design was the most appropriate way to appraise the predictive value of AAR without influencing the surgical indication. Moreover, at present, the decision to perform septoplasty remains primarily clinical because no universally accepted gold-standard diagnostic system has been established.

This study has limitations. First, the retrospective design limited the collection of patient-reported outcome measures (PROMs) and the availability of long-term follow-up data on sleep outcomes. Although the relationship between objective NAR values and PROMs remains debated, our primary focus was to assess the clinical relevance of AAR findings in a cohort of patients with already established nasal symptoms and a clear surgical indication. Secondly, this cohort may be affected by selection bias, as it includes only patients with OSAS who were referred for DISE combined with nasal surgery; therefore, it may not fully represent the broader OSAS population. Inclusion criteria were aimed at reducing selection bias due to intrinsic ethnic anatomical variations; nevertheless, they limited the generalizability of our findings to non-Caucasian populations. Power analysis confirmed the relatively limited sample size, highlighting the importance of future research including larger populations to strengthen those exploratory findings. The response to topical decongestion may vary across individuals, further reducing generalizability. Finally, single-center practice and measurement variability in rhinomanometry may limit broader relevance; however, the same equipment under uniform conditions was consistently used, and all examinations were performed by the same physicians.

Future studies should prospectively validate standardized AAR thresholds and assess their impact on surgical planning, patient-reported outcomes, and the need for staged procedures in OSA patients. Combining AAR with advanced 3D imaging or computational airflow models could refine the assessment of nasal obstruction and help with procedure selection. Also, integrated clinical–functional algorithms might help support more personalized surgical choices.

## 5. Conclusions

Preoperative AAR may help plan nasal surgery during DISE in patients with obstructive sleep apnea and nasal obstruction. Evaluating airflow resistance before and after decongestion can clarify the relative role of mucosal and structural factors and support a personalized choice between inferior turbinoplasty alone or combining it with septoplasty. Prospective studies are needed to confirm its predictive value and define practical thresholds for surgical planning.

## Figures and Tables

**Table 1:** General characteristics of the included population. DISE: Drug-Induced Sleep Endoscopy; sd: standard deviation; HSAT: Home Sleep Apnea Testing; AHI: Apnea–Hypopnea Index; ODI: Oxygen-Desaturation Index; SpO_2_: Oxygen saturation; CT90: Relative time of sleep spent with SpO_2_ < 90%.

Gender (%)	Male	35 (73)
	Female	13 (27)
Age (mean ± sd)		53 ± 11
Surgery (%)	DISE with turbinoplasty	27 (56)
	DISE with septoturbinoplasty	21 (44)
HSAT parameters (mean ± sd)	AHI	21.7/h ± 15
	ODI	21.8/h ± 15
	Mean SpO_2_	93.5% ± 2
	Minimum SpO_2_	81.0% ± 7
	CT90	8.9% ± 11
Upper airway comorbidities (%)	Non-allergic rhinitis	34 (67)
	Allergic rhinitis	13 (26)
	Asthma	2 (4)
	Chronic rhinosinusitis	11 (23)
	With nasal polyps	2 (4)
	Without nasal polyps	9 (19)

**Table 2:** Nasal airway resistance values. Results are expressed in Pa/cm^3^/s. Mean values ± standard deviation are displayed.

Basal	Right	Inspiratory	0.456 ± 0.680
		Expiratory	0.270 ± 0.310
	Left	Inspiratory	0.346 ± 0.329
		Expiratory	0.458 ± 1.493
	Total	Inspiratory	0.183 ± 0.154
		Expiratory	0.178 ± 0.130
Decongestion	Right	Inspiratory	0.229 ± 0.395
		Expiratory	0.158 ± 0.242
	Left	Inspiratory	0.227 ± 0.258
		Expiratory	0.340 ± 1.011
	Total	Inspiratory	0.103 ± 0.128
		Expiratory	0.134 ± 0.108

## Data Availability

Owing to privacy and ethical restrictions, the data presented in this study are available upon request to the corresponding author.
